# The structure-based cancer-related single amino acid variation prediction

**DOI:** 10.1038/s41598-021-92793-w

**Published:** 2021-06-30

**Authors:** Jia-Jun Liu, Chin-Sheng Yu, Hsiao-Wei Wu, Yu-Jen Chang, Chih-Peng Lin, Chih-Hao Lu

**Affiliations:** 1 The Ph.D. Program of Biotechnology and Biomedical Industry, China Medical University, Taichung, Taiwan; 2 Department of Information Engineering and Computer Science, Feng Chia University, Taichung, Taiwan; 3 Master’s Program in Biomedical Informatics and Biomedical Engineering, Feng Chia University, Taichung, Taiwan; 4 Graduate Institute of Biomedical Sciences, China Medical University, Taichung, Taiwan; 5 Yourgene Health, New Taipei City, Taiwan; 6 Department of Medical Laboratory Science and Biotechnology, China Medical University, Taichung, Taiwan

**Keywords:** Computational biology and bioinformatics, Structural biology

## Abstract

Single amino acid variation (SAV) is an amino acid substitution of the protein sequence that can potentially influence the entire protein structure or function, as well as its binding affinity. Protein destabilization is related to diseases, including several cancers, although using traditional experiments to clarify the relationship between SAVs and cancer uses much time and resources. Some SAV prediction methods use computational approaches, with most predicting SAV-induced changes in protein stability. In this investigation, all SAV characteristics generated from protein sequences, structures and the microenvironment were converted into feature vectors and fed into an integrated predicting system using a support vector machine and genetic algorithm. Critical features were used to estimate the relationship between their properties and cancers caused by SAVs. We describe how we developed a prediction system based on protein sequences and structure that is capable of distinguishing if the SAV is related to cancer or not. The five-fold cross-validation performance of our system is 89.73% for the accuracy, 0.74 for the Matthews correlation coefficient, and 0.81 for the F1 score. We have built an online prediction server, CanSavPre (http://bioinfo.cmu.edu.tw/CanSavPre/), which is expected to become a useful, practical tool for cancer research and precision medicine.

## Introduction

Single amino acid variation (SAV) refers to one amino acid substitution resulting from genetic polymorphisms. Nonsynonymous encoding variants alter the protein sequence. In extreme cases, this alteration affects the entire protein structure or function. The unique physicochemical properties of each type of amino acid means that the occurrence of the mutation in different positions of the sequence affects protein conformation and its function to different extents. It is vital to understand how the SAV influences protein and to clarify the links between genetic variations and human diseases. Most disease-related SAVs occur in structurally or functionally essential positions^1–3^. Previous research that mapped nonsynonymous SNPs to the structural surfaces of encoded proteins found that about 88% of disease mutations are located in the voids or pockets^4^. These mutation residues may affect the protein structure or the aggregation of the complex. Importantly, protein destabilization is a primary factor in many Mendelian diseases. Two-thirds of disease-related mutations adversely influence protein–protein interactions via loss of interactions, misfolding, or impaired expression^5,6^.

Structural dynamics are correlated to protein function, with evidence of missense-folding structures resulting in protein dysfunction^7,8^. If missense variants occur at the functional sites, the resulting changes in protein activity and binding affinity cause disease. SAVs located in the protein surfaces are also related to diseases, because these SAVs can destroy protein–protein interactions^9,10^. Increasing evidence indicates that SAVs are associated with several different cancers. Proteins containing harmful amino acid substitutions can affect pathways in different cancers^11–13^. Much evidence has revealed substantial changes in genomic sequences in patients with cancer. Ovarian cancer samples from The Cancer Genome Atlas (TCGA) project, for example, have revealed as many as 4,128 mutations at 575 genes in a cohort of 590 cases^14^. The TCGA project has also identified approximately 9,000 mutations at 575 genes among 564 patients with lung adenocarcinoma^15^. Recent research has suggested that somatic mutation accumulation is critical in tumorigenesis^16^. Moreover, while some variations may appear to be neutral, they may actually be driver mutations that contribute to cancer progression^17^. In the era of precision medicine, it is still difficult to precisely identify which genetic mutation serves as the trigger point of tumorigenesis without a systems network biology framework^18,19^. At the proteome level, amino acid substitutions caused by genetic codon transitions may explain the basis of human cancer^20^. Amino acid alterations appear to follow certain rules. For example, arginine has a positive charge that is important for balancing protein and DNA binding; however, arginine is highly mutated in various cancer types. The loss of arginine frequently influences the function of cancer-associated proteins, whereas gaining cysteine, an active and reducing agent, may enhance the capacity of a protein to neutralize reactive oxygen species (ROS) in the tumor environment^21–23^. Proteomic changes caused by proteins carrying missense mutations may help cancer cells adapt to environmental pressure^24^. While different types of cancers have unique properties that are not shared among all cancers, these different cancers may share some substitution patterns^25^. For instance, the amino acid substitution spectrum is similar in breast and digestive tract cancers, and is dominated by the alteration of glutamic acid to lysine^25^. Clarification of the relationship between SAVs and cancers using traditional experiments necessitates much time and many resources, so computational prediction methods are sorely needed in cancer biology research.

Machine learning has become a favored tool for data analysis, as this offers the capacity for leveraging big data and for analyzing the content of complex problems, clarifying information and content^26,27^. Many predictors have emerged that use machine learning as algorithms for SAVs. The two most important and commonly used categories of development strategies are the genetic-based and protein-based prediction systems. Several large-scale sequencing projects such as the TCGA project are widely utilized for genetic analysis^28,29^, some of which follow the American College of Medical Genetics and Genomics (ACMG) guideline^30^. The multifactorial variant prediction (MVP) is a genetic-specific multifactorial model that integrates 16 in silico predictors with the available clinical evidence^31^. However, while the genetic-based system has established a causal relationship between genes and cancer, this relationship is limited to cause and outcome. A comprehensive assessment of cancer driver mutation prediction models evaluated 33 commonly used prediction tools^32^, the top three (CHASM, CTAT-cancer and DEOGEN2) of which are cancer- or protein-based systems^33–35^; CHASM and CTAT-cancer are designed to incorporate the cancer protein, while DEOGEN2 predicts the deleteriousness of SAV by training the mutations in human inherited disease. Since the protein molecule is intimately involved in cellular processes, using the protein-based system to build a prediction system might be more informative for complex diseases such as cancer. However, although numerous prediction models have been developed, we lack the expertise for constructing accurate prediction tools for cancer and knowing which SAV descriptors to incorporate^36^.

We describe our development of a prediction model, which is capable of recognizing whether a particular SAV is cancer-related or is neutral. This model is not only able to discriminate physical changes for each SAV regarding protein function and structure, but it can also estimate how these changes contribute to cancer progression. We hypothesized that accounting for every kind of SAV might be a vital feature for cancer, so we designed a system that incorporates multiple prediction models and enables the user to extract critical features from cancer-related proteins. Our model provides a novel way forward for cancer research, not only for clinical outcomes but also for recognizing prognostic biomarkers, which we contend is a breakthrough for precision medicine.

## Materials and methods

### Dataset of SAVs

All SAV data were collected from CanProVar 2.0^37,38^, a human Cancer Proteome Variation database that stores both germline and somatic amino acid variations, including those related to the genesis or development of human cancers based on six sources, including the public databases HPI^39^, COSMIC^40^, OMIM^41^, and TCGA^42^, as well as two large-scale cancer genome resequencing studies^43,44^. CanProVar 2.0 contains 156,671 cancer-related SAVs from mutations that have been reported in cancer samples and 967,017 neutral SAVs from validated coding SNPs in the dbSNP database. In order to determine the exact protein structure of the SAV sequence, CanProVar 2.0 data were mapped to the proteins identified by BLAST^45^ from the Protein Data Bank. The search used six criteria, as follows: 1. The e-value of alignment results should be smaller than 1e−50; 2. The sequence identity of alignment results should be greater than 80%; 3. The alignment coverage of the protein structure should exceed 95%; 4. The organism of the aligned target protein should be *Homo sapiens*; 5. The experimental method ideally uses X-ray diffraction to extract the protein structure for aligning the target protein structures; and 6. The SAV position should be equidistant between the wild-type in the SAV sequence and the aligned target protein. After matching a protein with a 3-dimensional structure, CD-HIT^46^ was used to filter out homologous proteins. The CD-HIT cluster algorithm generates sets of protein families and uses the sequence identity cut-off of 0.3 to purify redundant proteins. Subsequently, the remaining 2,867 cancer-related SAVs and 7,562 neutral SAVs were our main training set, which were separated into 20 groups by using the representative wild-type amino acid of SAV. The numbers of cancer-related and neutral SAVs for each wild-type amino acid in the training set are listed in Table 1; δ is the cancer:neutral SAV ratio, which ranges from 0.2245 to 0.6693.

**Table 1:** The numbers of cancer-related and neutral SAVs for each wild-type (WT) residue and the total (bolded) in our training and independent datasets.

WT	Training set				Independent set 30		Independent set 40	
	Cancer	Neutral	Total	Ratio (δ)	Cancer	Neutral	Cancer	Neutral
ALA	187	657	844	0.2846	12	191	26	268
CYS	45	97	142	0.4639	6	21	9	36
ASP	216	412	628	0.5243	14	121	26	167
GLU	254	408	662	0.6225	11	114	27	174
PHE	85	127	212	0.6693	9	46	14	58
GLY	236	437	673	0.5400	11	129	21	171
HIS	77	188	265	0.4096	6	63	9	85
ILE	116	437	553	0.2654	5	113	9	178
LYS	98	263	361	0.3726	6	98	12	131
LEU	178	321	499	0.5545	6	99	17	141
MET	63	202	265	0.3119	2	56	12	83
ASN	90	340	430	0.2647	5	107	8	153
PRO	200	363	563	0.5510	9	100	24	134
GLN	83	212	295	0.3915	7	55	14	78
ARG	328	1,248	1,576	0.2628	14	335	30	459
SER	217	432	649	0.5023	12	131	25	176
THR	150	505	655	0.2970	7	146	13	203
VAL	156	695	851	0.2245	7	236	16	315
TRP	24	42	66	0.5714	1	14	3	22
TYR	64	176	240	0.3636	4	65	7	95
**TOTAL**	**2,867**	**7,562**	**10,429**	**0.3791**	**154**	**2,240**	**322**	**3,127**

Two independent sets were built, independent sets 30 and 40, which collected the proteins filtered out by CD-HIT in the previous step. Any proteins in the independent sets sharing more than 30 or 40% sequence identity with another protein in the same wild-type amino acid group of a training set were filtered out. The final analysis included 154 cancer-related SAVs and 2,240 neutral SAVs in the independent set 30, and 322 cancer-related SAVs and 3,127 neutral SAVs in the independent set 40. Table 1 lists the numbers of cancer-related and neutral SAVs for each wild-type amino acid in the independent sets 30 and 40. Typically, the 30% sequence identity was usually used as the criteria to remove homologous proteins. However, due to few cancer-related SAVs were collected in the independent set 30, we provide the extra independent set 40 for comparison.

### Prediction systems

We used the machine learning method to build two cancer-related SAV prediction systems. The first system, CanSavPre_w_, contained 20 individual prediction models constructed from 20 groups according to the SAV wild-type amino acid. Subsequently, the second prediction system, CanSavPre_wm_, divided the 20 groups into several subgroups by SAV mutated type. Those subgroups containing fewer than 30 SAVs were combined with other subgroups that had substitution scores exceeding zero based on BLOSUM62. Any subgroups that did not fit these criteria were omitted from the CanSavPre_wm_, construction and were instead included in the CanSavPre_w_ prediction models. The dataset was split into subgroups to determine the characteristics of specific wild-type amino acid alterations in each of the sequence-based, structure-based and microenvironment-based feature sets. As an illustration, a glycine would be built into prediction models containing for example acidic (e.g., aspartic or glutamic acid) and basic (e.g., arginine) mutated amino acids with distinct SAV features. Thus, our feature selection process effectively detects essential features. The second prediction system ultimately yielded 100 prediction models (see Supplementary Table S1).

Each prediction model was a two-level Support Vector Machine (SVM) classifier module. The SVM machine learning method is widely used for classifying protein structure or function in computational biology^47–52^. All SVM calculations were performed using LIBSVM (version 3.24)^53,54^, with the radial basis function (RBF) kernel. The first-level SVM comprised 12 SVM classifiers based on four repeats for three feature sets, which are described in the next section. All SAV descriptors in each feature set were fed into the SVM, and a five-fold cross-validation was performed during model training. The parameters (penalty and gamma values of the RBF kernel) were both trained by exponentially increasing the grid search from 2^–15^ to 2^15^ incorporating best values of informative measures.

The genetic algorithm (GA)^55,56^ was used to select features and optimize performance. The basic GA procedures are as follows: $N$ solutions ($S_{i} ,~i = 1, \ldots ,N$) are randomly generated as the starting population. Each solution $S_{i} ~$ is represented as a set of vectors $~S_{i} = \left( {\Phi ^{i} } \right)$. The feature vector $\Phi ^{i} ~$ is an $m$-dimensional vector, indicating the binary representations of $m$ features: If $f_{j}^{i} = 1$, the $jth$ feature is kept; if $~f_{j}^{i} = 0$, the feature $j^{{th}} ~$ is eliminated. In order to avoid any imbalance between positives and negatives in performance, four informative measures (Eqs. 1–4) for prediction performance were used as the fitness functions, consisting of accuracy (Acc), the Matthews correlation coefficient (MCC), the F1 score (F1), and summation of sensitivity and weighted specificity (Hybrid). They were calculated as follows:

1 $$Acc = \frac{{TP + TN}}{{TP + TN + FP + FN}},$$

2 $$MCC = \frac{{TP \times TN - FP \times FN}}{{\sqrt {\left( {TP + FP} \right)\left( {TP + FN} \right)\left( {TN + FP} \right)\left( {TN + FN} \right)} }}~,$$

3 $$F1 = \frac{{2 \times Precision \times Sensitivity}}{{Precision + Sensitivity}}~,$$

4 $$Hybrid = Sensitivity + \delta \times Specificity~,$$

where $Precision = \frac{{TP}}{{TP + FP}}$, $Sensitivity = \frac{{TP}}{{TP + FN}}$, $Specificity = \frac{{TN}}{{TN + FP}}$, *TP* represents true-positives, *TN* represents true-negatives, *FP* represents false-positives, *FN* represents false-negatives and $\delta$ is the ratio of the number of cancer-related SAVs to neutral SAVs, which are listed in Table 1.

In the initial population, $N$ solutions are randomly divided into two halves. $\alpha$ and $\beta$ have the best fitness in each half, and they are defined as $\alpha = \left( {\Phi ^{\alpha } } \right) = \max \left\{ {S_{1} , \ldots ,S_{{N/2}} } \right\}$ and $\beta = \left( {\Phi ^{\beta } } \right) = \max \left\{ {S_{{\frac{N}{2} + 1}} , \ldots ,S_{N} } \right\}$. In general, the three basic mechanisms driving the evolutionary processes in one generation consist of the selection, mutation and crossover processes.

#### Selection operator

In the $\tau ^{{th}}$ generation, the selection operators are defined as:

$$\alpha ^{\tau } = \max \left\{ {S_{1}^{{\tau - 1}} , \ldots ,S_{{N/2}}^{{\tau - 1}} ,\alpha ^{{\tau - 1}} } \right\},$$

$$\beta ^{\tau } = \max \left\{ {S_{{\frac{N}{2} + 1}}^{{\tau - 1}} , \ldots ,S_{N}^{{\tau - 1}} ,\beta ^{{\tau - 1}} } \right\}.$$

Note that for the special case of $\tau = 0$, $\alpha ^{0} ~$ and $\beta ^{0} ~$ are defined as 0. A new solution, $S_{i}^{\tau }$, is equal to $\alpha ^{\tau } ~$ if $i~$ is odd, while $S_{i}^{\tau } ~$ is equal to $\beta ^{\tau }$ if $i$ is even.

#### Mutation operator

We apply two types of mutation to the $N$ solutions $S_{i}$ s. In the case of $i = 1, \ldots ,N/2$, every $b$ bit of the vectors is subject to mutation: $b = \sim b$, if the mutation rate is less than a mutation threshold $\mu _{0} = 0.1$. In the case of $i = \frac{N}{2} + 1, \ldots ,N$, we randomly choose a bit from the vectors. These bits are then subject to mutation without any mutation thresholds.

#### Crossover operators

The crossover operations are carried out between $S_{{2p - 1}}$ and $S_{{2p}}$, where $p = 1, \ldots ,N/2$ and proceed as follows: one-point crossover is performed between $\Phi ^{{2p - 1}}$ and $\Phi ^{{2p}}$ if the crossover rate is less than the crossover threshold $\mu _{1} = 0.5$.

**Figure 1:**
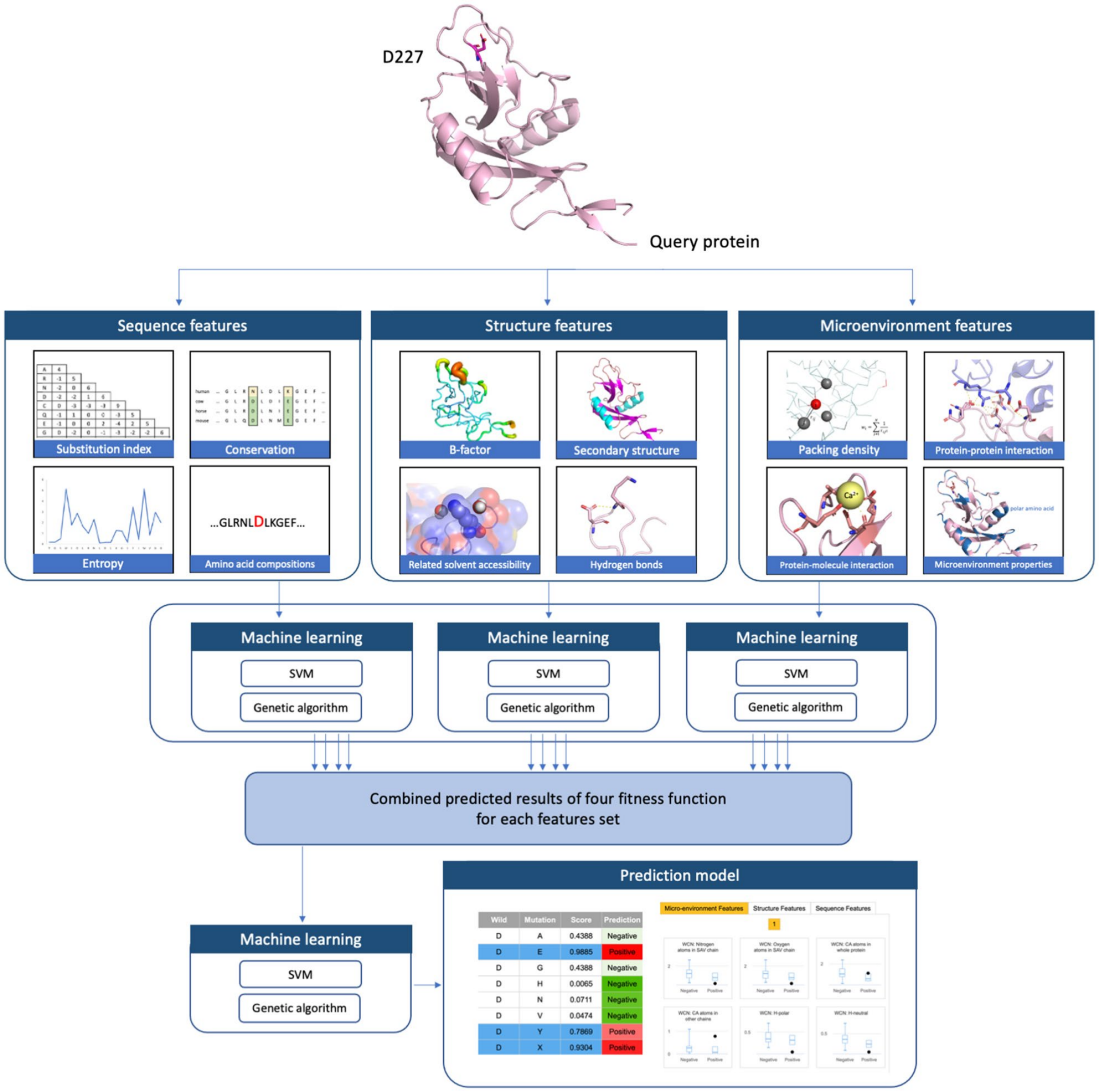
The workflow diagram represents the two-level SVM prediction system.

The second level of SVM classifiers is used to process the prediction results generated from 12 classifiers in the first level, to produce the final probability distribution of the relationship with cancer-related SAVs or neutral SAVs. The relationship with the largest probability is used as the final prediction. The two-level SVM system is shown schematically in Fig. 1.

### Classification of feature sets

SAV descriptors for machine learning were classified into three classes: sequence-based, structure-based, and microenvironment-based feature sets. For the sequence-based feature set, 44 descriptors were extracted from the protein sequence and approximately partitioned into three categories. The first category contained the most commonly used substitution index of wild-type SAV residues to mutations. Three kinds of substitution indices were used; the BLOSUM62^57,58^, PAM250^59^, and the position-specific scoring matrix (PSSM), which was derived from PSI-BLAST^60^. The second category represented the conservation for each residue compared with homologs. The 15 evolutional entropy values derived from PSI-BLAST were used to denote a sliding window containing 7 amino acids on either side of the SAV. The model also calculated average entropy values for window lengths 5 and 15, centered on the SAV, representing local- and wide-ranging sequence conservation, respectively. In the third category, an amino acid composition (AAC)^61^, a 15-residue peptide (with 7 amino acids on either side of the SAV), represented the composition of the neighboring residues. According to the physicochemical properties of residues, we used the following classification schemes^62^ of amino acid compositions: **H** for polar (RKEDQN), neutral (GASTPHY), and hydrophobic (CVLIMFW); **V** for small (GASCTPD), medium (NVEQIL), and large (MHKFRYW); **Z** for low (GASDT), medium (CPNVEQIL), and high polarizability (KMHFRYW); **P** for low (LIFWCMVY), neutral (PATGS), and high polarity (HQRKNED); **F** for acidic (DE), basic (HKR), polar (CGNQSTY), and nonpolar (AFILMPVW); **E** for acidic (DE), basic (HKR), aromatic (FWY), amide (NQ), small hydroxyl (ST), sulfur-containing (CM), aliphatic 1 (AGP), and aliphatic 2 (ILV). For clarity, these sequence-based descriptors are summarized in Table 2.

**Table 2:** List of descriptors in the sequence-based feature set.

#	Feature name	#	Feature name
1	Substitution index: BLOSUM62	23	AAC: *H*-hydrophobic (CVLIMFW)
2	Substitution index: PAM250	24	AAC: *V*-small (GASCTPD)
3	Substitution index: PSSM	25	AAC: *V*-medium (NVEQIL)
4	Entropy: 7th residue before SAV	26	AAC: *V*-large (MHKFRYW)
5	Entropy: 6th residue before SAV	27	AAC: *Z*-low polarizability (GASDT)
6	Entropy: 5th residue before SAV	28	AAC: *Z*-neutral (PATGS)
7	Entropy: 4th residue before SAV	29	AAC: *Z*-high polarizability (KMHFRYW)
8	Entropy: 3rd residue before SAV	30	AAC: *P*-low polarity (LIFWCMVY)
9	Entropy: 2nd residue before SAV	31	AAC: *P*-neutral polarity (PATGS)
10	Entropy: 1st residue before SAV	32	AAC: *P*-high polarity (HQRKNED)
11	Entropy: SAV	33	AAC: *F*-acidic (DE)
12	Entropy: 1st residue after SAV	34	AAC: *F*-basic (HKR)
13	Entropy: 2nd residue after SAV	35	AAC: *F*-polar (CGNQSTY)
14	Entropy: 3rd residue after SAV	36	AAC: *F*-nonpolar (AFILMPVW)
15	Entropy: 4th residue after SAV	37	AAC: *E*-acidic (DE)
16	Entropy: 5th residue after SAV	38	AAC: *E*-basic (HKR)
17	Entropy: 6th residue after SAV	39	AAC: *E*-aromatic (FWY)
18	Entropy: 7th residue after SAV	40	AAC: *E*-amide (NQ)
19	Entropy: Average of 15 residues	41	AAC: *E*-small hydroxyl (ST)
20	Entropy: Average of 5 residues	42	AAC: *E*-sulfur-containing (CM)
21	AAC: *H*-polar (RKEDQN)	43	AAC: *E*-aliphatic 1 (AGP)
22	AAC: *H*-neutral (GASTPHY)	44	AAC: *E*-aliphatic 2 (ILV)

The structure-based feature sets contained 13 descriptors extracted from PDB and DSSP^63,64^. The first structure-based descriptor used the B-factor value of the SAV ${\text{C}}\alpha$ atom; the B-factor value represents those atoms displaced from their mean positions in a crystal structure diminishes the scattered X-ray intensity. This displacement may be the result of temperature-dependent atomic vibrations, or static disorder in a crystal lattice. Our model also uses critical DSSP information regarding solvent accessibility and includes eight DSSP-defined elements in the secondary structure (i.e., H, B, E, G, I, T, S, and others), energy from the acceptor and donor backbone hydrogen bonds, and determines whether or not disulfide bonding exists. These structure-based descriptors are summarized in Table 3.

**Table 3:** List of descriptors in the structure-based feature set.

#	Feature name	#	Feature name
1	B-factor	8	Secondary structure: T
2	Related solvent accessibility	9	Secondary structure: S
3	Secondary structure: H	10	Secondary structure: Others
4	Secondary structure: B	11	Energy of backbone H-bond: acceptor
5	Secondary structure: E	12	Energy of Backbone H-bond: donor
6	Secondary structure: G	13	Disulfide bond
7	Secondary structure: I		

In the third feature set, the weighted contact number (WCN) model^65^ was used to describe the microenvironment properties of SAVs. This weighted contact number model has a local packing density profile, and research has reported a high correlation between the WCN profile and the sequence conservation profile^66^. The WCN value of atom $$i$$ was calculated by $$WCN_{i} = \mathop \sum \limits_{{j \ne i}}^{N} {\raise0.7ex\hbox{$1$} \!\mathord{\left/ {\vphantom {1 {r_{{ij}}^{2} }}}\right.\kern-\nulldelimiterspace} \!\lower0.7ex\hbox{${r_{{ij}}^{2} }$}}$$, where $$r_{{ij}}$$ was the distance between the atom $$i$$ and atom $$j$$, while $$N$$ was the number of calculated atoms. In this work, atom $$i$$ was defined as the $${\text{C}}\alpha$$ atom of SAV, and the different microenvironment properties were represented by calculated different atom types, or the source of atom $$j$$. The atom type of $$j$$ could be $${\text{C}}\alpha$$ atoms, nitrogen atoms or oxygen atoms of an amino acid. Moreover, the source of atom $$j$$ could be located within the same protein chain as SAV or the whole protein, representative of SAV packing density. Alternatively, the source could be derived from another protein chain or molecules such as DNA, RNA, ligands, or metal ions representing protein–protein or protein-molecule interactions. The packing density of SAV may be divided into different classifications representing the microenvironment properties wherein the SAV is located, such as polar, hydrophobic, acidic or basic, according to the physicochemical properties of residues containing the $${\text{C}}\alpha$$ atom *j*. The same classification schemes were used as described in the sequence-based feature set. The microenvironment-based descriptors are listed in Table 4.

**Table 4:** List of descriptors in the microenvironment-based feature set.

#	Feature name	#	Feature name
1	WCN: $C_{\alpha }$ atoms in SAV chain	17	WCN: *Z*-high polarizability (KMHFRYW)
2	WCN: Nitrogen atoms in SAV chain	18	WCN: *P*-low polarity (LIFWCMVY)
3	WCN: Oxygen atoms in SAV chain	19	WCN: *P*-neutral polarity (PATGS)
4	WCN: $C_{\alpha }$ atoms in whole protein	20	WCN: *P*-high polarity (HQRKNED)
5	WCN: Nitrogen in whole protein	21	WCN: *F*-acidic (DE)
6	WCN: Oxygens in whole protein	22	WCN: *F*-basic (HKR)
7	WCN: $C_{\alpha }$ atoms in other chains	23	WCN: *F*-polar (CGNQSTY)
8	WCN: Atoms in other molecules	24	WCN: *F*-nonpolar (AFILMPVW)
9	WCN: *H*-polar (RKEDQN)	25	WCN: *E*-acidic (DE)
10	WCN: *H*-neutral (GASTPHY)	26	WCN: *E*-basic (HKR)
11	WCN: *H*-hydrophobic (CVLIMFW)	27	WCN: *E*-aromatic (FWY)
12	WCN: *V*-small (GASCTPD)	28	WCN: *E*-amide (NQ)
13	WCN: *V*-medium (NVEQIL)	29	WCN: *E*-small hydroxyl (ST)
14	WCN: *V*-large (MHKFRYW)	30	WCN: *E*-sulfur-containing (CM)
15	WCN: *Z*-low polarizability (GASDT)	31	WCN: *E*-aliphatic 1 (AGP)
16	WCN: *Z*-neutral (PATGS)	32	WCN: *E*-aliphatic 2 (ILV)

## Results

### Performance evaluation

Table 5 compares the five-fold cross-validation performances of two prediction systems based on three different feature sets with the prediction performance of the second-level SVM, all of which are optimized by using MCC as the fitness function in GA. In our experiment, the individual prediction model using the sequence-based feature scheme outperformed the other two, while the performance of the model using the microenvironment-based feature was superior to that of the structure-based feature scheme. The outstanding performance of the combined model obtained in the second-level SVM procedure demonstrates that further information is very helpful for understanding and determining cancer-related factors.

**Table 5:** Comparisons of the five-fold cross-validation performance values in the training set of two prediction systems based on three feature sets, combined by second-layer SVM.

System	Feature set	Accuracy	Sensitivity	Specificity	MCC	Precision	F1 score
CanSavPre_w_	Sequence	0.7450	0.4350	0.8620	0.3202	0.5434	0.4832
	Structure	0.6995	0.3946	0.8146	0.2176	0.4454	0.4185
	Microenvironment	0.7184	0.3988	0.8391	0.2536	0.4832	0.4370
	Combined	0.7983	0.4356	0.9357	0.4452	0.7199	0.5428
CanSavPre_wm_	Sequence	0.8471	0.6292	0.9293	0.5978	0.7706	0.6928
	Structure	0.8022	0.4737	0.9262	0.4609	0.7078	0.5676
	Microenvironment	0.8311	0.5774	0.9268	0.5509	0.7487	0.6520
	Combined	0.8973	0.7837	0.9404	0.7382	0.8328	0.8075

All predictions were optimized using MCC as the fitness function.

**Figure 2:**
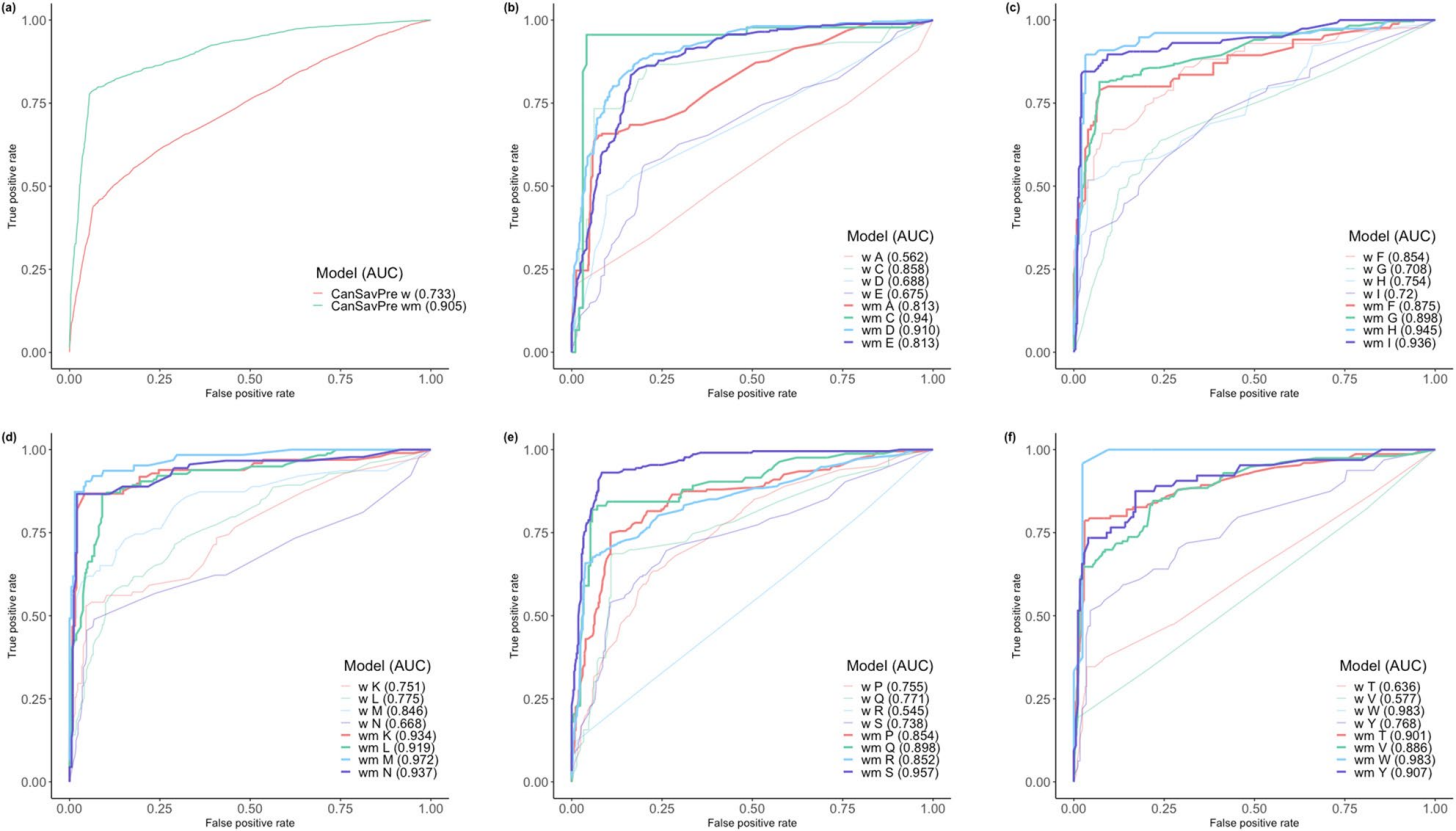
Training set ROC curves in two prediction systems. (**a**) Total groups in the two systems; (**b**) A, C, D, E groups; (**c**) F, G, H, I groups; (**d**) K, L, M, N groups; (**e**) P, Q, R, S groups; (**f**) T, V, W, Y groups.

Critically, CanSavPre_wm_ performed better than CanSavPre_w_ in all three individual feature sets and also in the combined set. Specific training and predicting models were built from the specific subgroups according to the wild-type and mutated types of SAV. Using a two-level SVM combining sequence-, structure- and microenvironment-based features, CanSavPre_w_ distinguished between SAVs that were or were not related to cancer, with an accuracy of 79.83%, a Matthews correlation coefficient of 0.45, and F1 score of 0.54. CanSavPre_wm_ is more effective, with an accuracy of 89.73%, a Matthews correlation coefficient of 0.74, and F1 score of 0.81. The fivefold cross-validation performance for each wild-type SAV of the CanSavPre_wm_ system is illustrated in Table 6. Figure 2 illustrates the ROC curve and compares the AUC values of each wild-type SAV in two systems.

**Table 6:** The five-fold cross-validation performance values in the training set of the CanSavPre_wm_ system for each wild-type (WT) residue and the total (bolded).

WT	TP	TN	FP	FN	Accuracy	Sensitivity	Specificity	MCC	Precision	F1 score
ALA	119	616	41	68	0.8709	0.6364	0.9376	0.6081	0.7438	0.6859
CYS	43	93	4	2	0.9577	0.9556	0.9588	0.9040	0.9149	0.9348
ASP	160	375	37	56	0.8519	0.7407	0.9102	0.6664	0.8122	0.7748
GLU	212	341	67	42	0.8353	0.8346	0.8358	0.6602	0.7599	0.7955
PHE	67	118	9	18	0.8726	0.7882	0.9291	0.7331	0.8816	0.8323
GLY	192	406	31	44	0.8886	0.8136	0.9291	0.7528	0.8610	0.8366
HIS	69	182	6	8	0.9472	0.8961	0.9681	0.8710	0.9200	0.9079
ILE	97	428	9	19	0.9494	0.8362	0.9794	0.8436	0.9151	0.8739
LYS	80	258	5	18	0.9363	0.8163	0.9810	0.8357	0.9412	0.8743
LEU	154	292	29	24	0.8938	0.8652	0.9097	0.7702	0.8415	0.8532
MET	55	199	3	8	0.9585	0.8730	0.9851	0.8835	0.9483	0.9091
ASN	78	333	7	12	0.9558	0.8667	0.9794	0.8643	0.9176	0.8914
PRO	150	324	39	50	0.8419	0.7500	0.8926	0.6512	0.7937	0.7712
GLN	68	200	12	15	0.9085	0.8193	0.9434	0.7714	0.8500	0.8344
ARG	216	1,189	59	112	0.8915	0.6585	0.9527	0.6538	0.7855	0.7164
SER	202	376	56	15	0.8906	0.9309	0.8704	0.7724	0.7829	0.8505
THR	118	490	15	32	0.9282	0.7867	0.9703	0.7907	0.8872	0.8339
VAL	97	681	14	59	0.9142	0.6218	0.9799	0.6912	0.8739	0.7266
TRP	23	41	1	1	0.9697	0.9583	0.9762	0.9345	0.9583	0.9583
TYR	47	169	7	17	0.9000	0.7344	0.9602	0.7356	0.8704	0.7966
**TOTAL**	**2,247**	**7,111**	**451**	**620**	**0.8973**	**0.7837**	**0.9404**	**0.7382**	**0.8328**	**0.8075**

### Case study: PI3K

**Figure 3:**
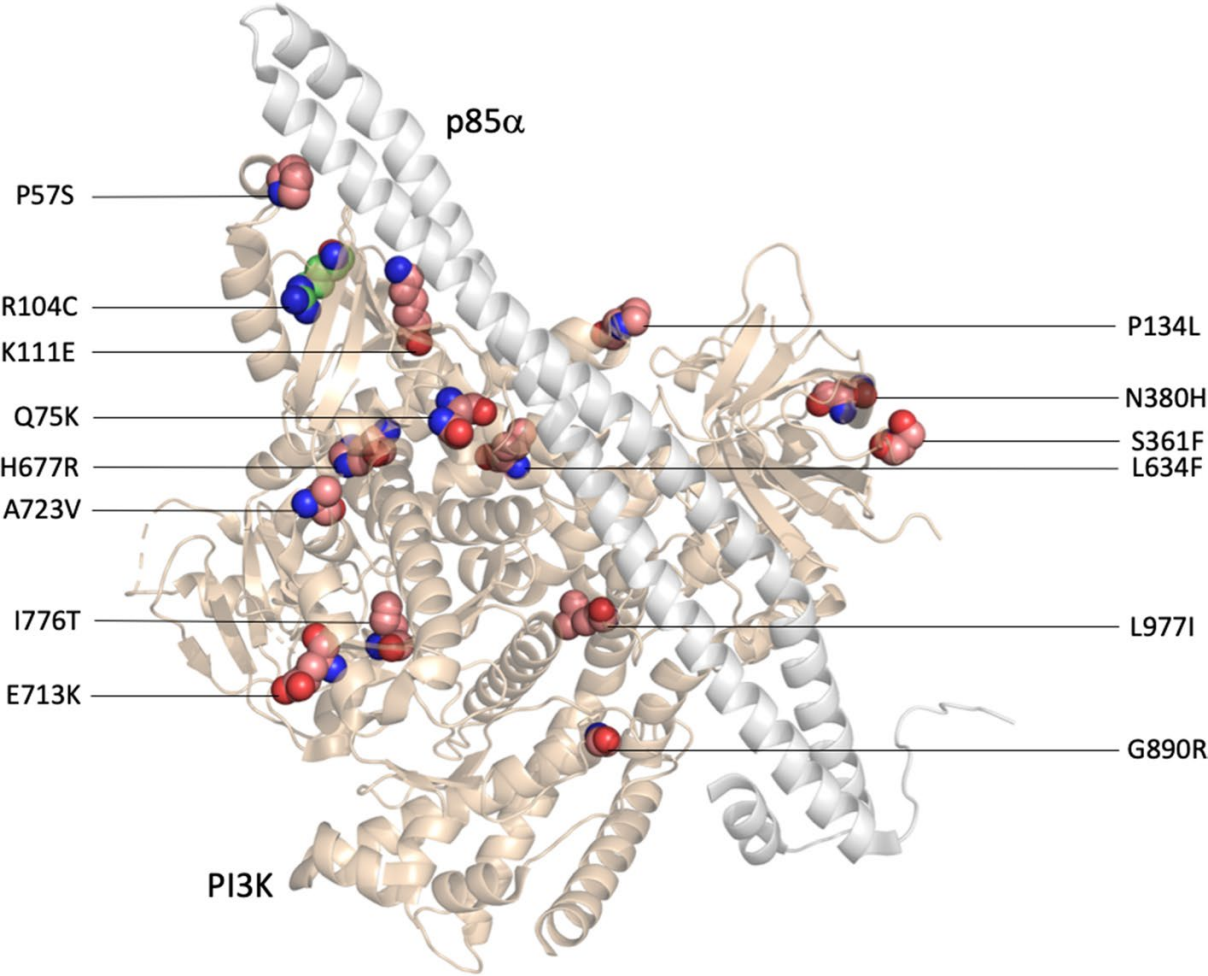
Protein structures of the PI3K/p85 $\alpha$ complex. The PI3K (wheat color) and p85 $\alpha$ (gray color) complex (PDB ID: 5DXU) in the cartoon is drawn by PyMOL^100^. ARG104 (illustrated by the green spheres) is a neutral SAV that mutates to CYS. The other residues shown in the pink spheres are all cancer-related SAVs.

The phosphatidylinositol-3-kinase (PI3K) signaling pathway contributes to several cellular processes, including metabolism, proliferation, differentiation and activation. Notably, the PI3K/AKT/mammalian target of rapamycin (mTOR) signaling pathway is one of the most important intracellular pathways and is also one of the most frequently dysregulated pathways in human cancers^67–70^. Several catalysis subunits exist for PI3K. Those that are encoded by PIK3cδ have been found to induce cell proliferation in colorectal cancer and other types of cancers^71,72^. The amino acid mutation of PI3Kcδ is closely related to oncogenic transformation, and numerous SAVs have been recorded as cancer-related in the COSMIC database, including P57S, Q75K, K111E, P134L, S361F, N380H, L634F, H677R, E713K, A723V, I776T, G890R, and L977I. Figure 3 illustrates the protein structures of PI3K and the p85 $\alpha$ complex (PDB ID: 5DXU)^73^; 14 amino acids, including one neutral and 13 cancer-related SAVs, are drawn as spheres. All SAVs, except H677R, are correctly predicted by our prediction system. It should be noted that another SAV, R104C, has been marked as a neutral SAV and is also predicted correctly. The predicted results of PI3K are listed in Table 7.

**Table 7:** Predicted results for the 14 SAVs of the PI3K.

SAV	Type	Score	Predicted results
P57S	Cancer-related	0.8590	TP
Q75K	Cancer-related	0.9973	TP
R104C	Neutral	0.1016	TN
K111E	Cancer-related	0.6590	TP
P134L	Cancer-related	0.7545	TP
S361F	Cancer-related	0.9408	TP
N380H	Cancer-related	0.9521	TP
L634F	Cancer-related	0.8879	TP
H677R	Cancer-related	0.0507	FN
E713K	Cancer-related	0.8438	TP
A723V	Cancer-related	0.8595	TP
I776T	Cancer-related	0.9656	TP
G890R	Cancer-related	0.9350	TP
L977I	Cancer-related	0.8000	TP

### Case study: D227Y of CD23

**Figure 4:**
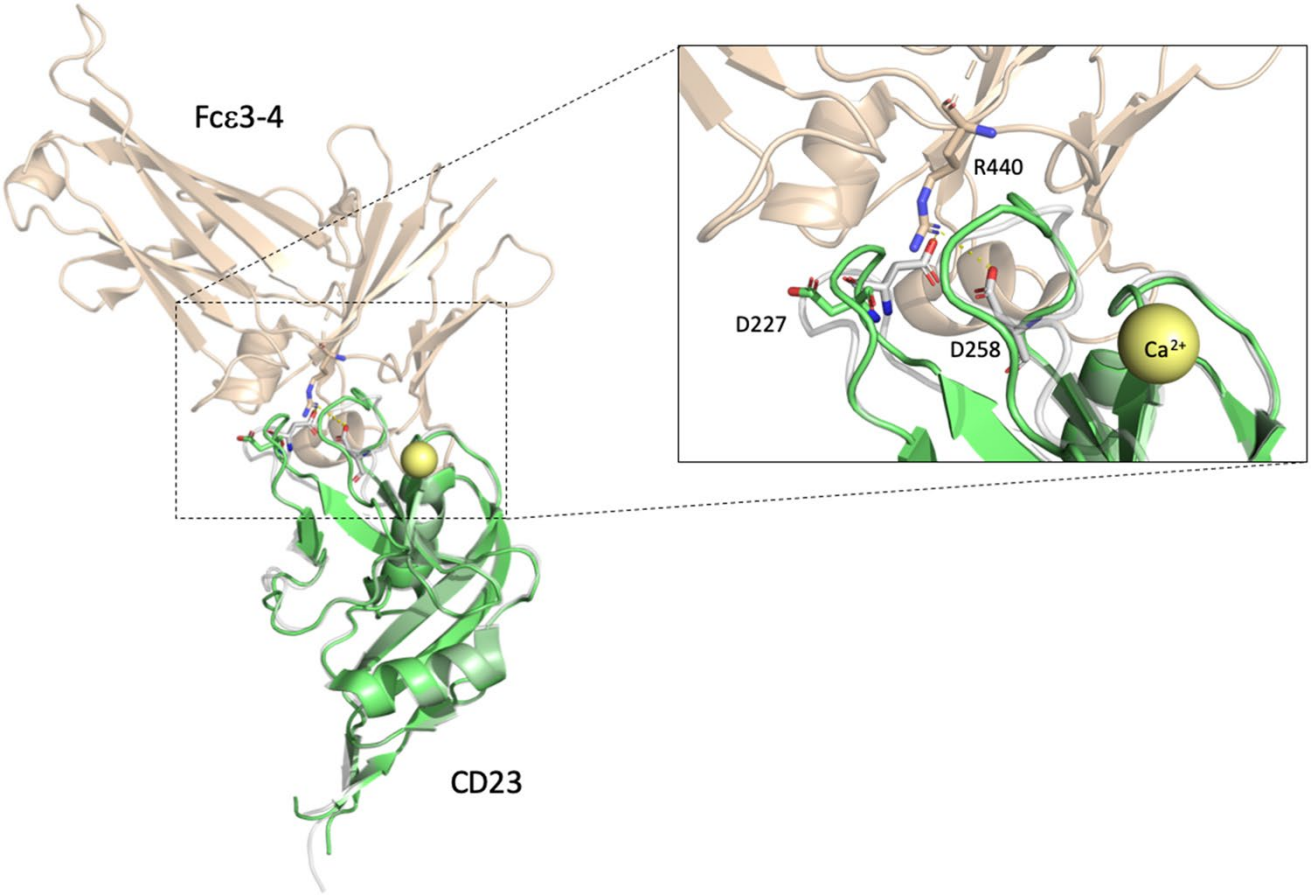
The superimposed structure containing CD23 apo and holo forms is obtained from the CD23 complex binding with Ca^2+^ and Fc$\varepsilon$ 3–4. The green cartoon represents the structure of the Ca^2+^-free wild-type CD23 lectin domain (PDB ID: 4G96)^83^. The structure of the CD23 holo form bound to Ca^2+^ complexed with Fc$\varepsilon$ 3–4 (PDB ID: 4GKO)^83^ is drawn in gray- and wheat-colored cartoons. Ca^2+^ is shown in a yellow bubble; the magnified view shows the interface of CD23 and Fc$\varepsilon$ 3–4. The D227 of the CD23 apo form is shown in the green stick. The salt bridges forming residues in the CD23 holo form and Fc$\varepsilon$ 3–4 complex are highlighted with sticks.

CD23 is the low-affinity receptor for IgE and is expressed on the surface of several hematopoietic cells^74^, such as lymphocytes^75^, monocytes^76^, follicular dendritic cells^77,78^, and bone marrow stromal cells^79^. Several stimuli regulate the expression of CD23, a critical factor for B-cell activation, growth, and IgE production (OMIM#151445). The D227Y mutation arising from an alteration of the *FCER2* gene has been reported in head and neck squamous cell carcinoma (HNSCC)^80^ and the colorectal neuroendocrine carcinoma mutational analyses project^81^. D227 is located in one of the conserved double-loops; the interface between CD23 and the carbohydrate protein, Fc$\varepsilon$ 3–4. Importantly, calcium (Ca^2+^) is a regulated ligand for CD23 binding affinity and Ca^2+^ binding enables loop1 and loop4 to change the conformation and increase the binding affinity. D227 (loop1) and D258 (loop4) form additional salt bridges between CD23 and Fc$\varepsilon$ 3–4^82,83^. Other bounds are involved in CD23 and Fc$\varepsilon$ 3–4 binding, while D227Y affects the binding affinity and IgE antitumor functioning (Fig. 4).

**Figure 5:**
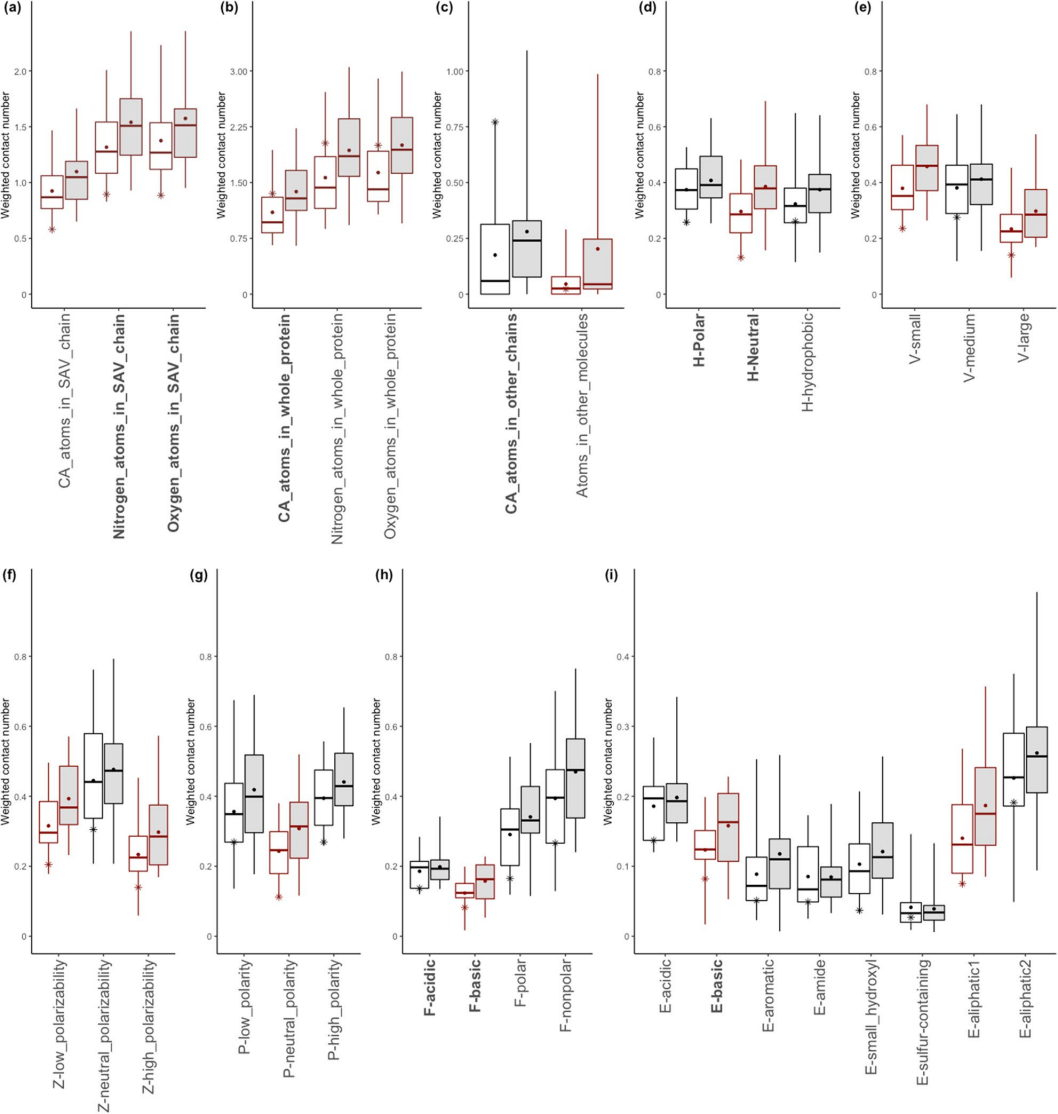
Boxplots of the microenvironment descriptors in the ASP that are altered to the TYR subgroup. All microenvironment descriptors are divided into nine groups: (**a**) atoms in the SAV chain; (**b**) atoms in whole protein; (**c**) atoms in other chains or molecules; (**d**) **H**-group; (**e**) **V**-group; (**f**) **Z**-group; (**g**) **P**-group; (**h**) **F**-group; and (**i**) **E**-group. The white and grey boxes represent the distribution of cancer-related and neutral SAVs. Red-framed boxes indicate that two sample *z*-testing revealed a significant difference between cancer-related and neutral SAVs, with a 95% confidence interval (z score > 1.96, p < 0.05). Labels of descriptors selected by the genetic algorithm are bolded in the *x*-axis. Star symbols denote the D227Y cases in CD23.

The boxplots of microenvironment descriptors in Fig. 4 depict the subgroup with the ASP to the TYR mutation. In several descriptors in Fig. 5, the distribution of cancer-related SAVs differs significantly from that of the neutral SAVs, with a 95% confidence interval by two sample *z*-test (z score > 1.96, p < 0.05). The z score is defined by $z = \frac{{\left( {x_{1} - x_{2} } \right) - \left( {\mu _{1} - \mu _{2} } \right)}}{{\sqrt {\frac{{\sigma _{1}^{2} }}{{n_{1} }} - \frac{{\sigma _{2}^{2} }}{{n_{2} }}} }}$, where $\left( {x_{1} - x_{2} } \right)$ and $\left( {\mu _{1} - \mu _{2} } \right)$ are the observed and expected differences between cancer-related and neutral SAVs, respectively. $\sigma _{1}$ and $n_{1}$ are the standard error and amount for the cancer-related SAVs group, and $\sigma _{2}$ and $n_{2}$ are for the neutral SAVs group. The cancer-related SAVs are located in the relatively low packing density region, encompassing ${\text{C}}\alpha$ atoms, nitrogen, or oxygen located in a single SAV chain, or in whole protein. D227Y in CD23 has a low WCN value in a single SAV chain but a relatively high WCN value in whole protein or other chains (Fig. 5a–c), because D227 is located in the interface of CD23 and Fc$\varepsilon$ 3–4, and is involved in their binding. Subsequently, the cancer-related SAVs have lower distributions of **H**-neutral (AGPHY), **V**-small (GASCTPD), **V**-large (MHKFRYW), **Z**-low polarizability (GASDT), **Z**-high polarizability (KMHFRYW), **P**-neutral polarity (PATGS), **F**-basic (HKR), **F**-nonpolar (AFILMPVW), **E**-basic (HKR), **E**-aromatic (FWY) and **E**-aliphatic1 (AGP) with the surrounding amino acids. This unique surrounding pattern is also found in the case of D227Y in CD23 (Fig. 5d–i).

### Case study: E194G of CASQ

**Figure 6:**
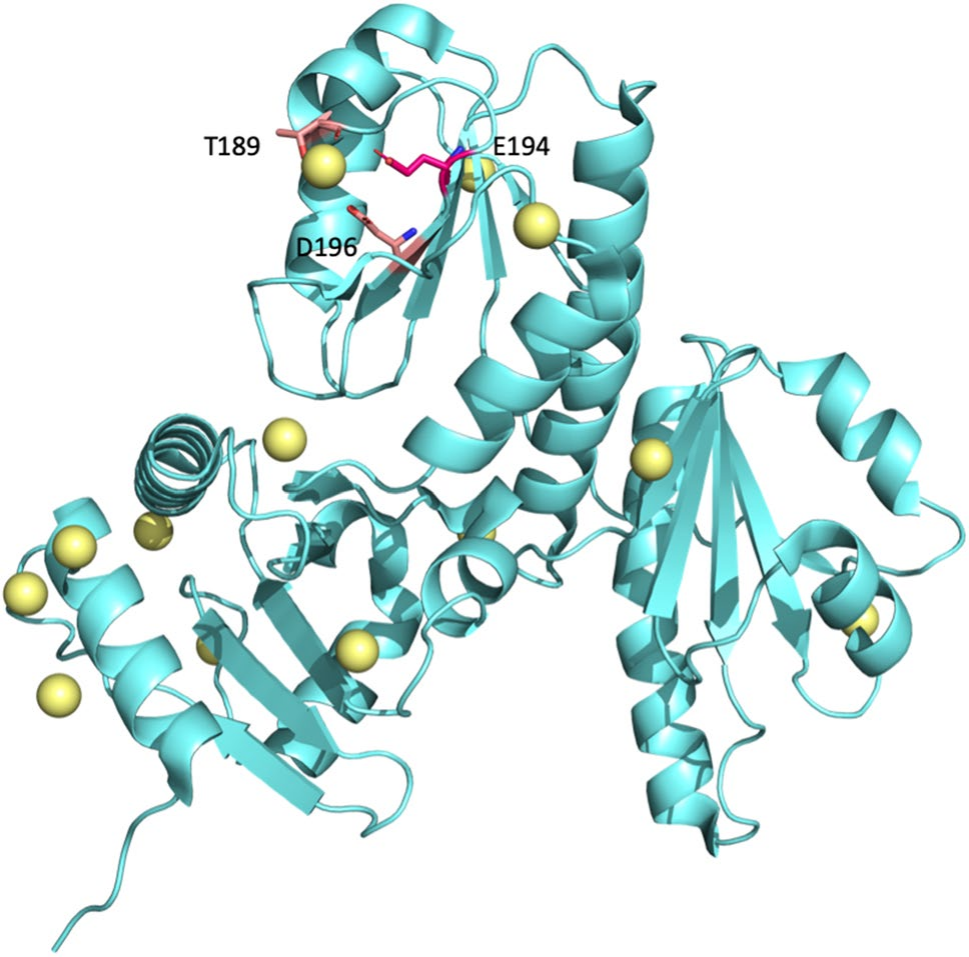
Protein structure of the human skeletal calsequestrin. The structure of CASQ (PDB ID: 3UOM)^89^ in the cyan-colored cartoon is drawn by PyMOL. All of the yellow bubbles are Ca^2+^ in CASQ. Three Ca^2+^ binding residues are highlighted with sticks in deep pink. E194G is a cancer-related SAV.

The calsequestrin (CASQ) is a Ca^2+^ buffering protein, capable of storing large amounts of Ca^2+^ in cardiac and skeletal muscles. Ca^2+^ is an essential molecule that can regulate diverse cellular processes, such as gene transcription, cell proliferation and migration^84–86^. Although most research into the CASQ has focused on cardiac muscle, CASQ in the Ca^2+^ signaling pathway is also vital in cancer research^87^, as this pathway is highly correlated with tumor growth and metastasis^88^. Importantly, T189, E194 and D196 can form a pack in the CASQ that harbors Ca^2+^^89^; this Ca^2+^ binding can be destroyed by the substitution E194G, causing the protein to lose its functionality (Fig. 6).

**Figure 7:**
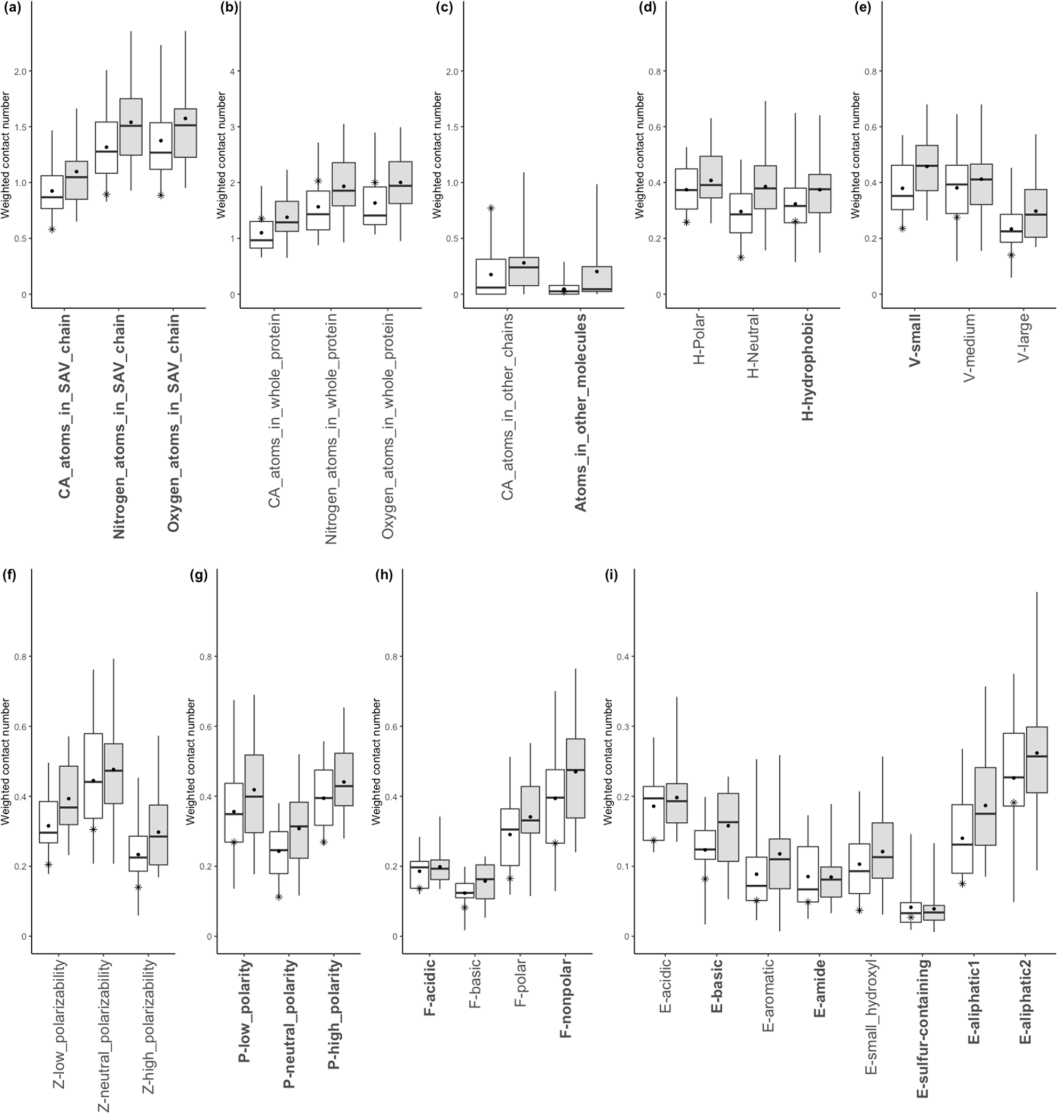
Boxplots of the microenvironment descriptors in the GLU that are altered to the GLY subgroup. All microenvironment descriptors are divided into nine groups: (**a**) atoms in SAV chain, (**b**) atoms in whole protein, (**c**) atoms in other chains or molecules, (**d**) **H**-group, (**e**) **V**-group, (**f**) **Z**-group, (**g**) **P**-group, (**h**) **F**-groups, and (**i**) **E**-groups. The white and grey boxes represented the distribution of cancer-related and neutral SAVs. The label of selected descriptors by the genetic algorithm are bold in the *x*-axis. The symbol stars denote the values of each feature in the case of E194G located at CASQ.

In the subgroup in which GLU mutates to GLY, none of the microenvironment descriptors of cancer-related or neutral SAVs reveal significant differences at the 95% confidence interval, although several relevant descriptors are found in the case of E194G in the CASQ. That is, E194 exhibits higher WCN values of oxygen in a single SAV chain, as well as higher WCN values of atoms in other molecules, due to the fact that the CASQ is GLU- and ASP-rich, as well as a Ca^2+^ buffering protein (Fig. 7a,b). Furthermore, higher WCN values were found in the microenvironment around E194 than in the third quartile of cancer-related SAVs in **H**-polar (RKEDQN), **V**-medium (NVEQIL), **Z**-low polarizability (GASDT), **P**-high polarity (HQRKNED), **F**-acidic (DE), and **E**-basic (HKR) descriptors and were lower than WCN values in the first quartile in **E**-sulfur-containing (CM) amino acid. Figure 7 depicts the microenvironment descriptor boxplots in the subgroup in which GLU mutates to GLY.

## Discussion

Many cancer-related tools are based on genetic or protein sequence information, because of limited protein structure information. Next-generation sequencing technology has led to the large-scale application of genomic information in cancer research and human health^90–93^. The crucial limitation of this information is that while the technology may determine tumor risk or recurrence, cause and effect remain undetermined. The launch of the Human Proteome Project (HPP) has enriched our understanding of the human proteome blueprint responsible for complex diseases, including cancer. Approximately 20,000 human cancer-related proteome studies have been recorded in PubMed since 2011^94^. However, it remains very difficult to construct a 3-dimensional protein structure from these proteins, which explains why the widely used cancer prediction tools incorporate sequences of genetic information or sequence information of proteins. Importantly, the spatial conformation of protein forms the functional unit.

**Figure 8:**
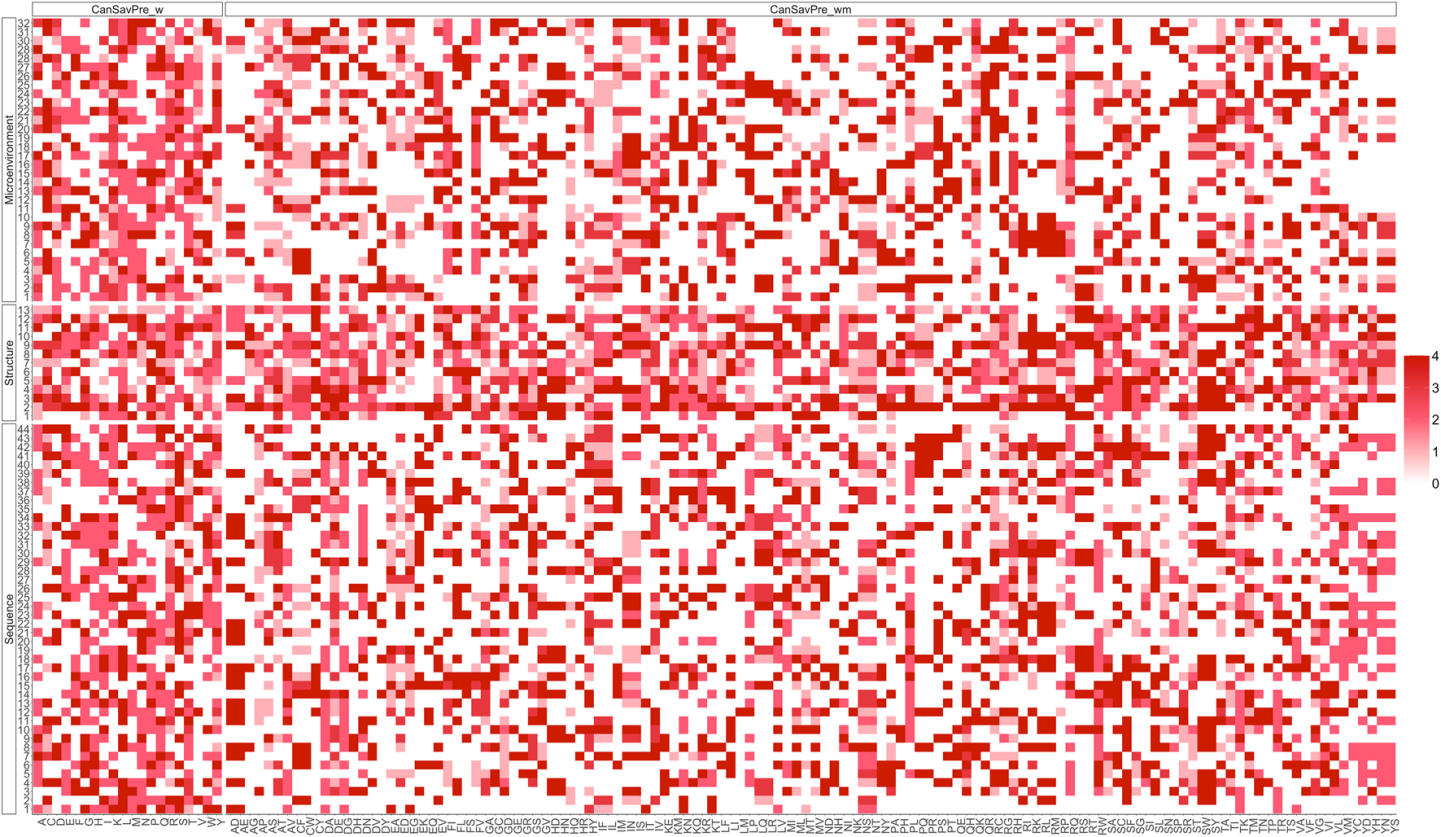
The heatmap of selected features in each training group of two prediction systems.

In this study, we describe how we developed a protein structure-based system, CanSavPre, to predict cancer-related single amino acid variations. Protein sequence and structure descriptors are used in the model training process. Our prediction system displays excellent performance, and its structural and microenvironmental properties enable us to observe mutating amino acids that generate malfunctioning proteins. Critical descriptors emerge through use of the feature selection procedure. Figure 8 shows the heatmap of selected features in each training group of two prediction systems. For each descriptor, the color represents how many times were selected during four training repeats with different fitness functions. Mutated residues and their functional impact can be characterized by analyzing the selected feature sets. Although further study is needed to reveal the cancer mechanism in most selected features, our results indicate that it is possible to reliably predict cancer-related SAVs.

We also found that it is essential to divide the training data into proper subsets, according to the wild-type and mutated SAVs. Each amino acid with its unique characteristics plays a different role in protein construction. The heatmap in Fig. 8 also indicates that the profiles of selected features in CanSavPre_wm_ are more legible than those in CanSavPre_w_. The resulting discrepancies influence changes in protein conformation activity, to different degrees. The data from our two-level prediction system optimize the outcomes from a system that uses only one level. However, the complexities of protein function in the extensive cellular networks necessitate critical information that identifies cancer-related SAVs.

The SAVs in the independent sets 30 and 40 were predicted using the CanSavPre_wm_ system and the prediction performance is illustrated in Table 8. Although a difference in prediction performance is evident between the independent sets and the training set, this is largely because the performance is optimized by a genetic algorithm in the training set. Our purpose was to extract significant features using an optimization procedure. Thus, performance in the training set is the upper bound of prediction. Another reason for the difference in prediction performance between sets might be too few cancer-related SAVs in the training and independent sets. In the training set, the average amount of cancer-related SAVs in the 100 subgroups of the CanSavPre_wm_ system is less than 30; the average amount is much smaller in the independent sets. The performance of independent sets might therefore be distorted, necessitating a greater number of cancer-related SAVs. Nevertheless, compared with DEOGEN2, which is mainly developed for deleterious amino acid variant prediction and publicly available, our prediction system performs better than DEOGEN2 in both two independent sets (Table 8).

**Table 8:** Comparison of prediction performance values with DEOGEN2 in the independent sets.

Method	Dataset	Accuracy	Sensitivity	Specificity	MCC	Precision	F1 score
CanSavPre	Independent set 30	0.8743	0.5844	0.8942	0.3419	0.2752	0.3742
CanSavPre	Independent set 40	0.8550	0.5839	0.8830	0.3699	0.3394	0.4292
DEOGEN2	Independent set 30	0.7222	0.5517	0.7337	0.1539	0.1225	0.2005
DEOGEN2	Independent set 40	0.6927	0.4693	0.7156	0.1170	0.1447	0.2212

Proteins are dynamic molecules with distinct, flexible structures that facilitate allosteric interactions between small molecules or proteins. For our prediction system, we focused on these interactions, i.e., physical contacts between proteins and molecules. A limitation of the crystal-protein structure is that it is capable of revealing only one condition of the protein–protein interaction. The critical purpose of a protein complex is to reflect interactions between single chain proteins. These interactions typically represent functional properties and are expected to be maintained through the genetic algorithm for feature selection. Importantly, technical limitations of the crystal structure prevent the formation of a protein complex in some conditions. Thus, some protein structures only include a part of the complex. These single chain proteins might restrict the range of our prediction system, because their conformations may differ from those of the protein complex. Our research seeks to define the phenomenon underlying cancer-related variations and how differences in conformations influence protein interactions, despite the limited available data on the protein complex.

Our case studies have used several different proteins to illustrate our protein structure-based system. In the first example, the PI3K protein family is well recognized for its association with cancer. The strict rule for our data extraction means that CD-HIT, the cluster database, filters out the homologous proteins and avoids weighting the mutation residues for the training model. Thus, the isoform PI3Kc**δ** is selected as the representative isoform, even though PI3Kca is one of the most readily recognized isoforms in cancer research^95,96^. The most frequent and pathogenic mutation residues recorded in the TCGA, E542K and E545K, are predicted correctly in our system (see Supplementary Fig. S1). The evidence suggests that these two hotspot mutations induce glycolysis in cervical cancer cells via the β-catenin/SIRT3 signaling pathway^97^. Both glutamic acid mutations (E542K and E545K) are located in the helical domain. Biochemical studies have demonstrated that these mutated residues interact with p85a, so the alteration may affect the inhibitory activity of p85a^98^.

Our prediction system also provides observations of the microenvironment for the SAV residues. The other case studies discussed in our results illustrate how the feature processes cope with the descriptors such as, for example, how the feature selection process manages ASP alteration patterns. This process also yields critical detail regarding ASP alterations (Fig. 4). ASP is a polar amino acid with a negative charge and the carboxyl group in the side chain of aspartic acid allows it to accept hydrogen atoms. Our model calculates the area surrounding the ASP. When the area surrounding the cancer-related ASP is unoccupied space, our model observes fewer basic amino acids compared with neutral ASP environments. This implies that, in this situation, the ASP might assume an interaction role with protein, whereby an ASP mutation can lead to an abnormal protein and different binding activity. This might explain how cellular changes result in tumor activity, as is illustrated in our second case study. In contrast, GLU is an amino acid with high polar residues and a negative charge that is also involved in the hydrogen atom acceptor role, with a distinctly different pattern to that of ASP (Fig. 6). Cancer-related GLUs are located in a denser region within a hydrophobic environment. Although ASP and GLU share similar characteristics, their different microenvironments justify construction of the prediction modules.

We suggest that protein structure and microenvironment features are the cornerstones of cancer research. Slight alterations in protein conformation properties may change the functional activity of molecular interactions. Clarifying protein structure is critical to understanding how a protein mutation can lead to cancer. However, the critical necessity for the protein structures is also the limitation of our prediction systems. Many emerging computational methods are attempting to clarify protein structure. For those proteins that only have sequence information, scientists can use a homology modeling method or an artificial intelligence system to extract the details needed for structural information. Notably, AlphaFold2 is considered to be an excellent solution to the problematic issue of protein folding^99^. As more protein structures become available over time, our model will benefit from the enriched databases, regardless of whether the data are sourced from experimental or predicted methods.

In conclusion, we have developed a structure-based cancer-related single amino acid variation prediction system. This system not only displays excellent performance, but it also observes how the amino acid substitution influences protein activities. The descriptors provided by our system may offer targets for further research. Moreover, performance is markedly enhanced by the fact that the model includes conformation properties and details of the microenvironments surrounding SAVs. Furthermore, our algorithm detects the best combination of feature vectors for examining specific amino acid variations. Importantly, our model is a user-friendly web-based tool that scientists will find extremely useful when performing cancer research and precision medicine, particularly when investigating rare tumor mutations. The many genetic mutations in human cancers offer us numerous targets and possibilities that may be incorporated into our model, emphasizing its importance in cancer research.

## Supplementary Information

Supplementary Information.
