# Supplementary material for: The structure-based cancer-related single amino acid variation prediction
Source: Sci Rep. 2021 Jun 30;11:13599. doi: 10.1038/s41598-021-92793-w (PMC8245468; doi:10.1038/s41598-021-92793-w)
Supplement: Supplementary file 1 — Supplementary Information. [file 41598_2021_92793_MOESM1_ESM.docx]

**SUPPORTING INFORMATION**

**SUPPLEMENTARY TABLE 1** List of 100 sub-groups for each wild amino acid type.

| **Wild type** | **Mutated type** | **Number of sub-group** |
| --- | --- | --- |
| A | DE, G, P, S, T, V | 6 |
| C | FW, Y | 2 |
| D | AG, E, H, N, V, Y | 6 |
| E | AG, D, K, Q, V | 5 |
| F | IV, L, S | 3 |
| G | A, C, D, E, R, S, V | 7 |
| H | DN, Q, R, Y | 4 |
| I | FL, M, NS, T, V | 5 |
| K | E, MQ, NT, R | 4 |
| L | F, I, M, P, QR, V | 6 |
| M | I, LV, T | 3 |
| N | D, HY, I, K, ST | 5 |
| P | A, H, L, QR, S, T | 6 |
| Q | E, H, K, R | 4 |
| R | C, G, H, ILM, K, P, Q, ST, W | 9 |
| S | AG, C, FWY, I, L, N, P, R, T | 9 |
| T | A, I, KR, M, N, P, S | 7 |
| V | A, F, G, I, L, M | 6 |
| W |  | 0 |
| Y | C, DNS, H | 3 |
| **ALL** |  | **100** |

**
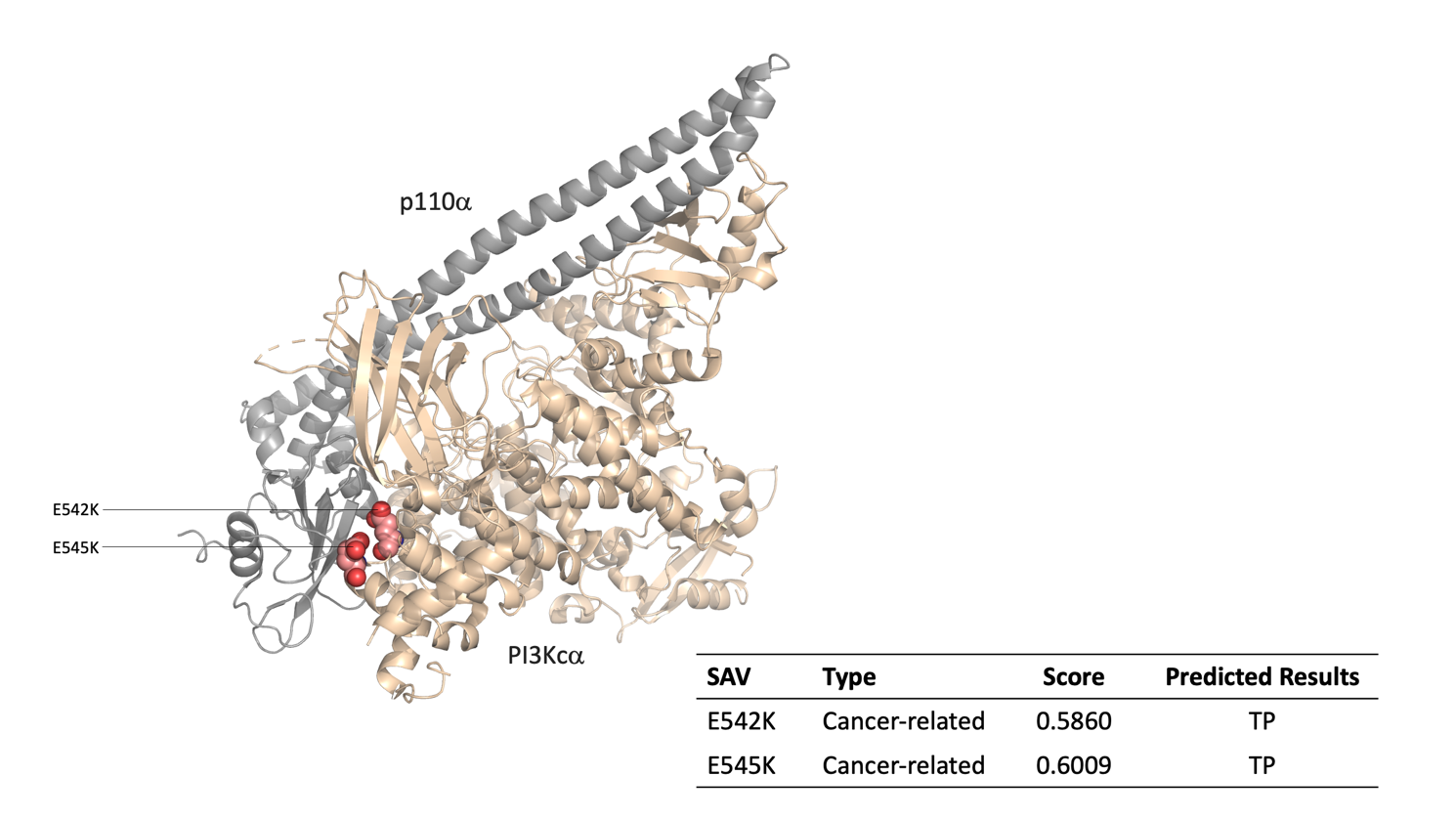
 SUPPLEMENTARY FIGURE 1 Protein structure of PI3K and p110**$\boldsymbol{\alpha}$ **complex.** The PI3K (wheat color) and p110$\alpha$ (gray color) complex (PDB ID: 5XGH) in the cartoon was drawn by PyMOL [[92](#_ENREF_92)]. The residues shown in the pink spheres are cancer-related SAVs. And the table shows the predicted results for the PI3K.
